# Structural and calorimetric studies demonstrate that the hepatocyte nuclear factor 1β (HNF1β) transcription factor is imported into the nucleus via a monopartite NLS sequence

**DOI:** 10.1016/j.jsb.2016.06.018

**Published:** 2016-09

**Authors:** Mareike M. Wiedmann, Shintaro Aibara, David R. Spring, Murray Stewart, James D. Brenton

**Affiliations:** a Department of Chemistry, University of Cambridge, Lensfield Road, Cambridge CB2 1EW, UK; b Cancer Research UK Cambridge Institute, University of Cambridge, Li Ka Shing Centre, Robinson Way, Cambridge CB2 0RE, UK; c MRC Laboratory of Molecular Biology, Francis Crick Avenue, Cambridge Biomedical Campus, Cambridge CB2 0QH, UK

**Keywords:** CCC, ovarian clear cell carcinoma, HNF1β, hepatocyte nuclear factor 1β, HNF1β^DBD^, HNF1β DNA binding domain, NLS, nuclear localization signal, IBB, Importin-β-binding domain, mImportin-α, mouse Importin-α, xImportin-α, *Xenopus* Importin-α, GST, glutathione S-transferase, Importin-α, Nuclear import pathway, Nuclear localisation signal sequence (NLS), Hepatocyte nuclear factor-1β (HNF1β), Site-directed mutagenesis, X-ray crystallography, Isothermal titration calorimetry

## Abstract

The transcription factor hepatocyte nuclear factor 1β (HNF1β) is ubiquitously overexpressed in ovarian clear cell carcinoma (CCC) and is a potential therapeutic target. To explore potential approaches that block HNF1β transcription we have identified and characterised extensively the nuclear localisation signal (NLS) for HNF1β and its interactions with the nuclear protein import receptor, Importin-α. Pull-down assays demonstrated that the DNA binding domain of HNF1β interacted with a spectrum of Importin-α isoforms and deletion constructs tagged with eGFP confirmed that the HNF1β ^229^KKMRRNR^235^ sequence was essential for nuclear localisation. We further characterised the interaction between the NLS and Importin-α using complementary biophysical techniques and have determined the 2.4 Å resolution crystal structure of the HNF1β NLS peptide bound to Importin-α. The functional, biochemical, and structural characterisation of the nuclear localisation signal present on HNF1β and its interaction with the nuclear import protein Importin-α provide the basis for the development of compounds targeting transcription factor HNF1β via its nuclear import pathway.

## 1 Introduction

Ovarian clear cell carcinoma (CCC) accounts for 5–10% of ovarian cancer cases (Anglesio et al., 2011, Kato et al., 2008). Prognosis for patients with advanced stage or for relapsed disease is poor because of intrinsic resistance to platinum based chemotherapy and the lack of targeted therapies available (Tan et al., 2013, Tan and Kaye, 2007), although Bitler et al. (2015) have recently discovered a way of targeting cancers with *ARID1A* mutations by targeting EZH2 methyltransferase activity. Common mutations in CCC include loss of function mutations in the chromatin remodeling gene *ARID1A* in 46–57% of cases (Jones et al., 2010, Wiegand et al., 2010), activating mutations in *PIK3CA* (Kuo et al., 2009) in 33–46% of cases, and loss of *PTEN* in 20% of cases (Anglesio et al., 2011, Landen et al., 2008, Tan and Kaye, 2007). Overexpression of the HNF1β transcription factor is the most important clinical immunohistochemical marker for the disease because it is ubiquitously overexpressed in CCC, both at the mRNA and protein level (Hirotaka Kajihara et al., 2010, Kato et al., 2006, Tsuchiya et al., 2003, Yamaguchi et al., 2010). In CCC the *HNF1B* gene is upregulated by hypomethylation of its CpG island whereas, in high grade serous ovarian cancer, HNF1β expression is silenced via hypermethylation (Kato et al., 2008, Shen et al., 2013a), suggesting that HNF1β has a loss of function (tumour suppressor) role in high grade serous ovarian cancer but a gain of function (oncogenic) role in CCC (Gounaris et al., 2011, Shen et al., 2013b). This hypothesis is supported by the observation that nearly half of the overexpressed genes identified in CCC are downstream targets of HNF1β (Kobayashi et al., 2009, Yoshida et al., 2009). Evidence that targeting HNF1β might have utility was provided by Liu et al. (Liu et al., 2009) who showed that downregulation of HNF1β increased cisplatin- and paclitaxel-mediated cytotoxicity.

Transcription factor HNF1β (also known as vHNF1, vAPF, LF-B3 and Tcf2) is expressed in the liver, digestive tract, pancreas and the kidneys, where it plays a crucial role in early differentiation (Lu et al., 2007). Sequence-specific DNA binding is mediated by a bipartite motif that consists of a POU homeodomain (POU_H_) and a POU specific domain (POU_S_) (Rosenfeld, 1991, Ryan and Rosenfeld, 1997). HNF1β has 70% sequence homology to HNF1α and both proteins are atypical members of the POU transcription factor family and bind DNA as both homo- and heterodimers (Bach et al., 1991, Rey-Campos et al., 1991). Human HNF1β is constructed from three domains: the dimerization domain, which is further stabilised by the dimerization cofactor of HNF1 (DcoH), the transactivation domain, which is involved in binding transcriptional co-activators [15], and the POU DNA binding domain (HNF1β^DBD^).

Transcription factors together with histones, DNA polymerase, RNA polymerase and many other proteins, have specific amino acid sequences, termed nuclear localisation signals (NLSs), that are recognised by members of the karyopherin family that facilitate their nuclear import (reviewed by Lange et al., 2007). Many NLS sequences are recognised in the cytoplasm by a heterodimeric transport carrier complex composed of Importin-β (also known as Karyopherin-β1) and Importin-α (reviewed by Stewart, 2007). Nuclear pore complexes (NPCs) are the channels through which macromolecules, such as proteins and RNA, are transported between the cytoplasm and nucleus (reviewed by Stewart, 2007). Small molecules and proteins (<40 kDa) can pass through NPCs by passive diffusion, but larger proteins require carriers to overcome the NPC physical barrier. The autoinhibitory Importin-β binding (IBB) domain of Importin-α (Kobe, 1999) binds to Importin-β in the cytoplasm, enabling classical NLSs (cNLS) to bind to Importin-α either via a major site, a minor site, or both (Fontes et al., 2000, Lange et al., 2007). There are two types of cNLS that are recognised by Importin-α that consist of either a single cluster (monopartite) or two clusters (bipartite) of positively charged residues, primarily lysines or arginines, that assume an ordered state once bound by Importin-α (reviewed by Lange et al., 2007, Marfori et al., 2011, Marfori et al., 2012). Monopartite cNLSs are exemplified by the simian virus 40 large T-antigen (SV40) NLS ^126^PKKKRRV^132^ (Lange et al., 2007). The cargo:carrier heterotrimer is then translocated into the nucleus in an energy dependent manner powered by RanGTPase (reviewed by Stewart, 2007). In the nucleus, RanGTP then binds Importin-β leading ultimately to the release of the cargo.

To function, HNF1β needs to be translocated to the cell nucleus and so we have investigated and characterised the putative NLS that has been proposed to lie between the two POU domains of HNF1β. This putative NLS was identified in domain swapping experiments and studies of nephrogenesis, in which truncated GFP-HNF1β fusion constructs retaining the POU_H_ domain showed exclusive nuclear localisation in transfected HeLa cells (Bohn et al., 2003, Wu et al., 2004). However, the precise location of the HNF1β NLS has not been defined. Because of the potential importance of HNF1β as a target in diseases such as CCC, we have identified and characterised extensively its NLS and its interactions with Importin-α. We demonstrate that the sequence ^229^KKMRRNR^235^ in HNF1β^DBD^ is responsible for the nuclear import of the protein. Several eGFP-constructs of HNF1β were generated and Importin-α binding of the HNF1β^DBD^ was assessed by both pull-down experiments and ITC. We also determined the crystal structure of the HNF1β NLS peptide bound to Importin-α. The identification and structural characterisation of the HNF1β NLS and its interaction with the nuclear import protein Importin-α provides a basis for the development of inhibitors targeting the nuclear import of transcription factor HNF1β along the lines suggested by Stelma et al. (2016).

## 2 Materials and methods

### 2.1 Mammalian cell culture

HEK293T cells were cultivated in Dulbecco’s Modified Eagle medium (DMEM) (1X) supplemented with 5% foetal bovine serum (FBS) (Invitrogen) and 0.5% penicillin/streptomycin (P/S). CCC cell lines PEO1, JHOC5, JHOC7, JHOC9, OVISE and SKOV3 cells were grown in RPMI 1640 medium (1X) supplemented with 10% FBS and 1% P/S. Normal Ovarian Surface Epithelial (IOSE) cells were cultivated in NOSE-CM: MCDB 105/medium 199 (1:1 ratio, Sigma Aldrich), 15% FBS, 10 ng/ml EGF (Invitrogen), 0.5 μg/ml hydrocortisone (Sigma Aldrich), 5 μg/ml insulin (Sigma Aldrich), 34 μg protein/ml BPE (Invitrogen). All cell lines were maintained at 37 °C in 5% CO_2_ and were mycoplasma tested on a regular basis (Biorepository Core, CRUK CI, Cambridge). Cell counts were conducted using a Vi-CELL Cell Viability Analyzer.

### 2.2 Protein extraction from mammalian cells

Cell pellets were washed with phosphate buffered saline (PBS) and 200 μl protein lysis buffer (50 mM Tris pH 8.0, 150 mM NaCl, 5 mM EDTA, 0.5% Igepal, to which 2 tablets/100 ml of protease inhibitor cocktail tablet (Roche)) was added. The mixtures were incubated on ice for 30 min, lysed by syringing four times using a 26 G needle, and centrifuged at 14,800*g* for 10 min at 4 °C. Protein concentrations were measured using the DirectDetect IR spectrometer (Merck Millipore) according to the manufacturer’s instructions.

### 2.3 Western blotting

Denatured protein extracts were separated using NuPAGE Novex 4–20% Tris-Glycine gels and transferred to a Millipore Immobilon FL PVDF membrane (Invitrogen). Primary antibodies were used as follows: goat anti-HNF1β (sc-7411, polyclonal, Santa Cruz Biotechnology, 1:1000) and rabbit anti-GAPDH (G9545, 14C10, 1:5000, Cell Signalling Technology). An Odyssey Infrared Imaging System (Li-Cor) and associated secondary antibodies: donkey anti-goat (800) (1:15,000) and donkey anti-rabbit (800) (1:5000) were used to detect material. The expression levels observed with different cells were evaluated using GraphPad Prism version 6.0 for Mac, GraphPad Software, La Jolla California USA, www.graphpad.com. Analysis of variance rejected the null hypothesis that the expression levels were equal (P < 0.002) and Šídák’s multiple comparison modification of Student’s *t*-test was used to evaluate the significance of the levels seen with different cells relative to that seen with the control HGSOC cell line.

### 2.4 Confocal microscopy: eGFP imaging and immunofluorescence assay

For immunofluorescence (IF), cells were fixed with 4% paraformaldehyde in PBS for 10 min at room temperature, rinsed in Tris buffered saline (TBS) for 2 × 5 min and permealized in TBS-0.5% Triton X-100 for 10 min. Fixed cells were then rinsed in TBS-0.1% Triton X-100 for 3 × 3 min and blocked in 10% goat serum in TBS for 30 min. HNF1β was stained with anti-HNF1β (SAB1406512, mouse polyclonal, 1:300, Sigma Aldrich) overnight at 4 °C. Cells were washed with TBS-0.1% Triton X-100 (Fisher Scientific) for 3 × 5 min and the secondary antibody (AlexaFluor 568, goat anti-mouse IgG (H + L) 1:1000, Invitrogen) was added and incubated for 60 min diluted in antibody dilution solution consisting of TBS-0.1% Triton X-100, 2% Bovine Serum Albumin (BSA) (Cell Signaling Technology) and 0.1% sodium azide. All experiments included an unstained control, a “secondary only” control, and a negative control using the PEO1 cell line. Cells transduced with eGFP-HNF1β, the HNF1β ^229^KKMRRNR^235^ deletion mutant, or the control eGFP constructs, were fixed with 4% paraformaldehyde in PBS for 10 min at room temperature, then rinsed in TBS for 2 × 5 min. Nuclei were stained using DAPI (1 μg/ml in TBS) for 10 min. Cells were washed in TBS-0.1% Triton X-100, rinsed in TBS and stored in TBS-0.1% Triton X-100. For microscopy imaging, cover slips were drained, mounted and sealed using Prolong Gold (Invitrogen) and glass slides (Thermo Specific). Slides were left to dry overnight at room temperature in the absence of light and were then stored at 4 °C. Cells were imaged using a Leica tandem confocal microscope.

### 2.5 Site directed mutagenesis to generate HNF1β NLS deletion construct

Lv103 (EX-F0366-Lv103, Genecopoeia) is a lentiviral transfer vector containing an eGFP-HNF1β fusion coding sequence. Lv105 (EX-EGFP-Lv105, Genecopoeia) only contains the eGFP coding sequence. The plasmids were confirmed by sequencing (GATC, Konstanz, Germany) before and after mutagenesis (Lv103 Fw: 5′-CCGACAACCACTACCTGA-3′; Rv: 5′-ATTGTGGATGAATACTGCC-3′ and Lv105 Fw: 5′-ATCCACGCTGTTTTGACC-3′; Rv: 5′-AATACTGCCATTTGTCTCG-3′).

Mutagenesis experiments were conducted using the Q5 site directed mutagenesis kit (NEB). The ^229^KKMRRNR^235^ deletion construct was generated using mutagenesis primers (Fw: 5′-TTCAAATGGGGGCCGC-3′; Rv: 5′-GTTGGTGGGCTCAGAGCAG-3′). Plasmids were then transformed in *E. coli* (One Shot® Stbl3™, Invitrogen) and streaked on Amp-containing agar plates. Single colonies were picked and tested for plasmid containing colonies by PCR. Plasmids were extracted by Plasmid Mini Prep (Qiagen) and quantified (Qubit).

### 2.6 Lentivirus production and transduction

The general protocol devised by Cribbs et al. (2013) was used for lentivirus production. The transfer vectors Lv103, Lv105, the ^229^KKMRRNR^235^ deletion mutant, and plasmids pRVS-Rev, pVSV-G, and p-MDLg-pRRE were verified by restriction digest. For each transfection sample, 16 μg transfer vector, 10.4 μg pMDL/pRRE, 4 μg pRSV-Rev and 5.6 μg pVSV-G were used. After 24 h, expression of GFP protein was observed in the GFP control virus HEK293T sample. Virus containing supernatant was harvested posttransfection according to the protocol by Kutner et al. (2009). The number of transducing units (TU) was determined by flow cytometry analysis with GFP as the reporter protein. For titration, 1 × 10^5^ cells per well were seeded in a 12-well plate and 0.5 ml DMEM with 5% FBS added. Virus of respective concentration was pipetted over the HEK293T cells. Duplicate virus dilutions of 1:500, 1:1000, 1:2000, 1:5000 were added. An untreated control was included. The medium was changed 24 h posttransfection. 72 h posttransfection cells were harvested by trypsinization. Cells were collected by centrifugation at 500*g* for 5 min at room temperature. The supernatant was discarded and cell pellets were resuspended in PBS. A green fluorescent protein propidium iodide (GFP PI) based assay for flow cytometric measurement of transfection efficiency and cell viability was performed (LSR-II machine). The titre was calculated from dilutions that gave 1–40% GFP-positivity and averaged subsequently using the following formula:

$$\text{Titre}\left(\frac{\text{TU}}{\text{ml}}\right)=\frac{(\text{frequency of GFP-positive cells})\times \text{no}.\text{cells plated}\times \text{dilution factor}}{\text{volume of inoculum}}$$

where, frequency of GFP-positive cells is the percentage of cells that are positive for GFP divided by 100 (acceptable range: 0.01–0.40), dilution factor is the dilution of the virus stock used and volume of inoculum is the total volume transduced. The titre was calculated to be $2.26\times 10^{8}\frac{\mathrm{TU}}{\mathrm{ml}}$.

For transduction of the PEO1 cell line, 1 × 10^6^ cells per well were seeded in a 12-well plate to which 0.5 ml of the respective medium was added. Virus was added so that the desired multiplicity of infection (MOI) was obtained. Polybrene 0.5–10 μg/ml was added to increase transduction efficiency. Transduction efficiencies were determined by flow cytometry analysis using an LSR-II machine. For selection of transduced PEO1 lines, a puromycin kill curve was constructed (data not shown) to determine the optimum concentration of puromycin required to kill all untransduced cells while minimising toxicity effects in transduced cells. A puromycin concentration of 0.1 μg/ml was determined which was used in all experiments.

### 2.7 Quick change mutagenesis to generate GST-tagged HNF1β^DBD^ and mImportin α1 mutants

The cloning of the DNA binding domain of HNF1β into the pGEX-TEV plasmid was conducted as described by Lu et al. (2006). The plasmid was confirmed by sequencing by Source Bioscience (Cambridge, UK) using PGEX5 and PGEX3 primers (Source Bioscience).

### 2.8 Protein expression in bacterial cells and purification

All proteins were expressed in *E. coli* BL21 (DE3) CodonPlus-RIL cells using IPTG induction over 18 h at 18 °C. The cells were harvested by centrifugation and resuspended in 50 mM Tris/HCl pH 8.0, 500 mM NaCl, 5 mM DTT for GST-tagged constructs or 50 mM Tris/HCl pH 8.0, 500 mM NaCl, 20 mM Imidazole pH 8.0 for His_6_-tagged proteins. The *E. coli* were lysed by two passes through an Emulsiflex C3 system (AVESTIN) running at a pressure of 15,000 psi and the lysates clarified by centrifugation at 48,000*g*. Either Ni-NTA agarose resin (QIAGEN) or glutathione Sepharose 4B resin (GE Healthcare) was added to the clarified lysate containing His_6_-tagged proteins or GST-tagged proteins. The resin was pooled and packed into a gravity filtration column and washed extensively with their respective lysis buffers. 1 mg of TEV protease (purified in-house) was added to the washed resin and incubated for 18 h at 4 °C. The tag-free protein of interest was then collected from the flow-through and subjected to the next purification step. HNF1β^DBD^ was purified further by heparin affinity chromatography using a HiTrap Heparin HP column (GE Healthcare Life Sciences). HNF1β^DBD^ was eluted from the column using a 0.1–1 M NaCl gradient. All proteins were purified to homogeneity by size exclusion chromatography using a HiLoad™ 26/60 Superdex™ 75 prep grade column (GE Healthcare) pre-equilibrated in 20 mM HEPES buffer pH 8.0, 200 mM NaCl. Fractions containing pure and homogenous proteins identified by SDS-PAGE analysis were pooled and concentrated using an Amicon Ultra 15 centrifugation filtration unit. Protein concentrations were measured using a Nanodrop (Thermo) microspectrophotometer and small aliquots of the protein were flash frozen in liquid nitrogen and stored at −80 °C until required.

### 2.9 GST pull-downs

After bacterial cell lysis, cell pellets expressing the two proteins of interest were mixed and pull-down experiments were conducted as described above for glutathione affinity chromatography and were visualised by SDS-page analysis.

### 2.10 Isothermal calorimetry

The K_d_ of mImportin-α1 with the HNF1β NLS peptide (Ac-TNKKMRRNRFK-NH_2_, purchased from Insight Biotechnology) was determined by isothermal titration calorimetry (ITC) using a MicroCal™ iTC200 (GE Healthcare Life Sciences). The protein and peptide samples were made up in the identical buffer (20 mM Hepes pH 8, 200 mM NaCl). 350 μl of protein sample (0.02 mM) and 200 μl of ligand (0.2 mM) were prepared for each experiment. Sample concentrations were confirmed by amino acid analysis (PNAC Facility, Biochemistry Department, Cambridge University). All experiments were conducted at 25 °C. The data was analysed using the Origin™ Software (MicroCal) using a one site binding model.

### 2.11 Protein crystallography

Diffraction quality crystals of the Importin-α1^ΔIBB^:HNF1β^NLS^ complex were obtained by sitting drop vapour diffusion where 200 nl of 10% PEG 8 K, 0.09 M NPS, 20% ethylene glycol, 0.1 M Buffer 1 (Morpheus Screen, Molecular Dimensions) was mixed with 200 nl of Importin-α1^ΔIBB^:HNF1β^NLS^. The Importin-α1^ΔIBB^:HNF1β^NLS^ complex was produced by mixing Importin-α1^ΔIBB^ and HNF1β^NLS^ peptide (Ac-TNKKMRRMRFK-NH_2_, Insight Biotechnology) at a 1:1.1 M ratio and a concentration of 6.7 mg/ml.

Crystals were cryoprotected in the mother liquor supplemented with 20% glycerol and cooled by plunging into liquid nitrogen. X-ray diffraction data were collected on beamline I03 at the Diamond Light Source (Didcot, UK). Reflections were indexed and integrated using *DIALS* as implemented in *Xia2* (Kabsch, 2010) and then scaled and merged in *AIMLESS*, ensuring a completeness of >98% in the outermost shell while maintaining CC_1/2_ > 0.3 (Evans and Murshudov, 2013). The structure was solved by molecular replacement using *Phaser* with the structure of ΔIBB-mImportin-α1 complexed with a minor site small molecule inhibitor (PDB ID: 4U54 – Holvey et al., 2015) to avoid introducing model bias into the NLS binding sites. Iterative cycles of rebuilding using *COOT* (Emsley et al., 2010) and refinement using *PHENIX* (Adams et al., 2010) were used to generate the final model (Table 1) that had an R-factor of 18.4% (R-free = 22.0%) and a MolProbity (Chen et al., 2010) score of 0.95 (100th percentile).

## 3 Results and discussion

### 3.1 HNF1β is overexpressed in CCC cell lines

**Fig. 1:**
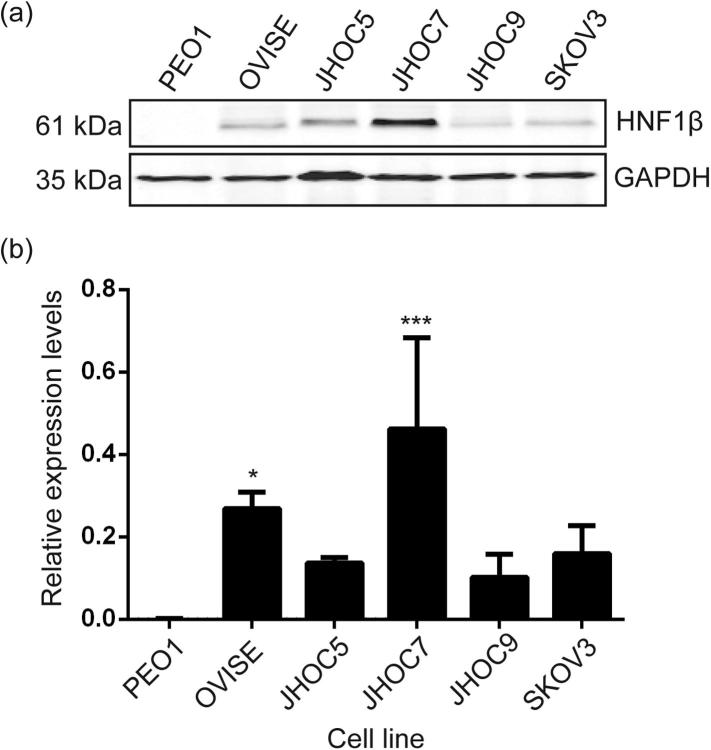
(A) HNF1β protein expression levels in CCC cell lines OVISE, JHOC5, JHOC7, JHOC9, SKOV3 and a negative control HGSOC cell line, PEO1; (B) Data is the mean (n = 3, three biological replicates) and SEM. Expression levels were normalised to the housekeeping protein GAPDH. The expression levels observed with JHOC5 (P < 0.005, ∗∗∗) and OVISE (P < 0.05, ∗) cells were significantly higher than PEO1.

HNF1β protein expression in different CCC cell lines was determined by Western blotting (Fig. 1) using the HGSOC cell line PEO1 (which does not express HNF1β) as a negative control. The CCC lines OVISE, JHOC5, JHOC7, JHOC9 and SKOV3 cell lines were used as models of CCC that overexpress HNF1β. Several splice variants of HNF1β are known to contain different isoforms of the C-terminal domain that is responsible for activation of transcription. These isoforms act as transdominant repressors (Bach and Yaniv, 1993). Only the 61 kDa isoform of HNF1β was detected in our CCC cell lines (Fig. 1) (Bach and Yaniv, 1993, Tsuchiya et al., 2003). HNF1β expression levels varied considerably between these cell lines. Analysis of variance of the data in Fig. 1 rejected the null hypothesis that the expression levels were equal (P < 0.002) and the higher level seen in JHOC7 compared with PEO1 cells was significant at the 0.5% level (using Sidak’s modification for multiple comparisons), consistent with hypomethylation of the HNF1β CpG island (Kato et al., 2008, Kato et al., 2007).

### 3.2 Identification of the HNF1β nuclear localisation signal

**Fig. 2:**
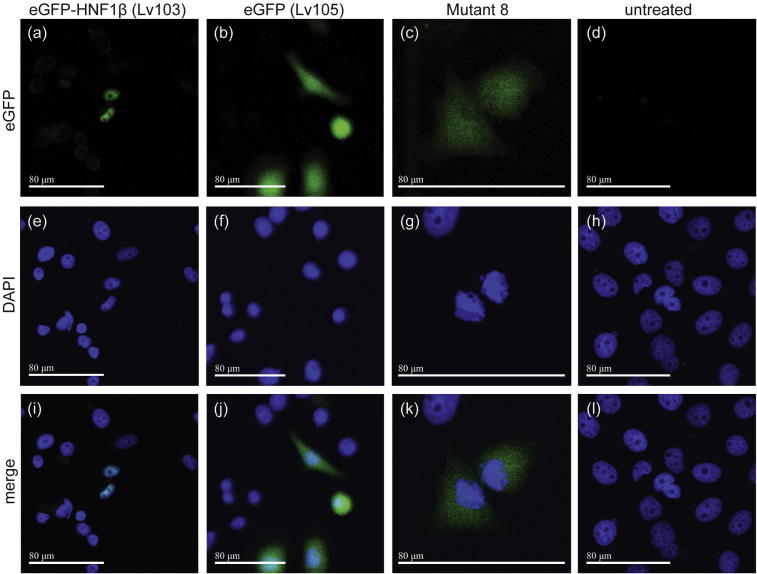
eGFP imaging of PEO1 transduced lines with (a) eGFP-HNF1β (Lv103) showed primarily nuclear localisation of the GFP signal, whereas cells transduced with eGFP (Lv105) alone (b) showed both nuclear and cytoplasmic localisation. In cells transduced with eGFP-HNF1β (Lv103) in which the seven-residue NLS had been deleted (c, Mutant 8) the eGFP signal was mislocalized to the cytoplasm. The scale bar represents 80 μm. Images were taken on a Leica tandem confocal microscope using 20×, 40× and 60× objectives.

To confirm the identity of the NLS, the PEO1 cell line was transduced with lentiviruses Lv103 (eGFP-HNF1β), Lv105 (eGFP) and the ^229^KKMRRNR^235^ deletion mutant. In cells transduced with eGFP-HNF1β, the fusion protein (eGFP-HNF1β (Lv103) – Fig. 2A (Mutant 8)) was primarily nuclear, whereas cells transduced with Lv105 (eGFP) showed nuclear and cytoplasmic localisation of the eGFP protein (eGFP (Lv105) – Fig. 2B). Deletion of the seven-residue ^229^KKMRRNR^235^ NLS sequence in HNF1β resulted in its becoming mislocalized to the cytoplasm (Mutant 8, Fig. 2C), confirming the importance of this sequence for the nuclear import of HNF1β. HNF1α has an analogous KKGRRN sequence that differs only at residue 231, where Met is changed to Gly (Wu et al., 2004) and which probably also functions in a similar manner. Interestingly, all transduced cells (that were selected using optimised puromycin concentrations) were apoptotic within 24 h. We speculate that re-expression of a transcription factor that is epigenetically silenced in HGSOC cell line PEO1 (Shen et al., 2013a) may have a negative effect on proliferation, but further work is necessary to determine the effects of re-expression.

### 3.3 HNF1β^DBD^ binds primarily to the major site on Importin-α

**Fig. 3:**
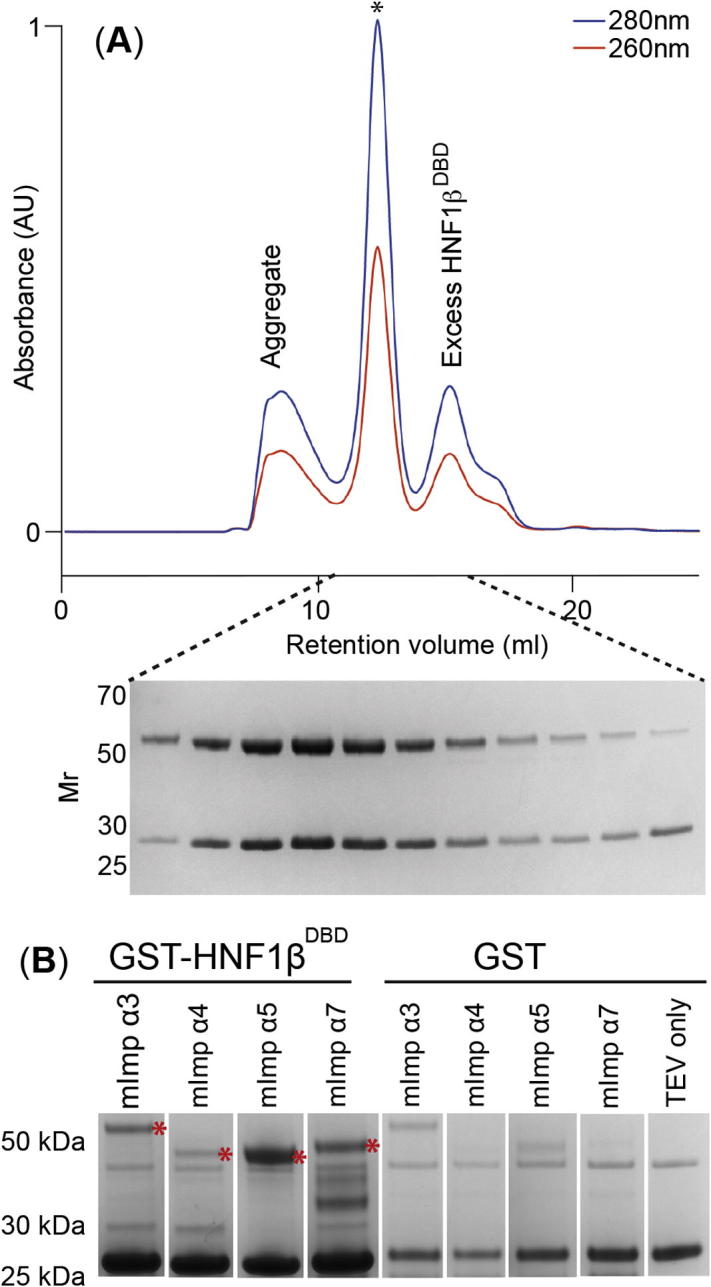
(A) Stable complex formation between ΔIBB mouse Importin-α1 (50 kDa) and HNF1β (27 kDa) during size exclusion chromatography. Fractions were analysed by Coomassie stained SDS-PAGE (∗ marks the peak for the complex); (B) GST pull-down with liberated HNF1β showing pull-downs using different ΔIBB mouse Importin-α isoforms (red ∗).

**Fig. 4:**
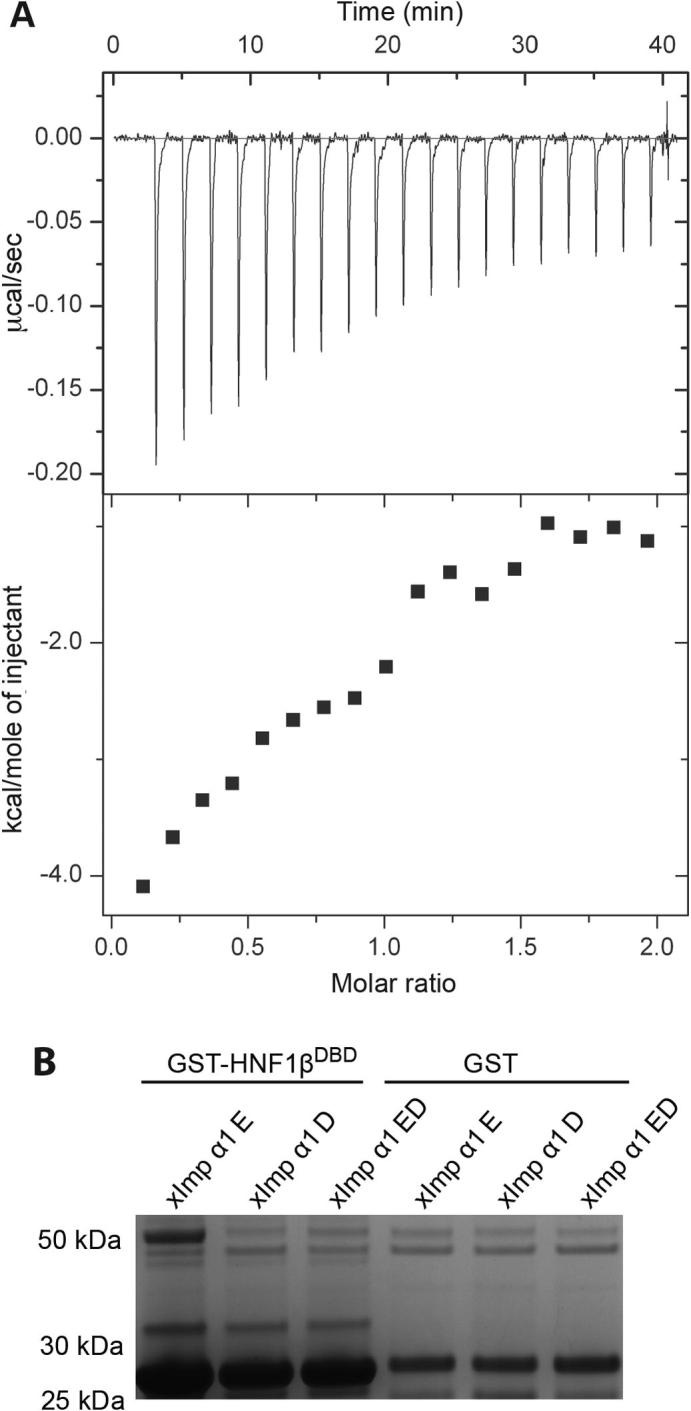
(A) ITC titration for the binding of the HNF1β NLS peptide to ΔIBB mImportin-α1. Fitting the data (with χ^2^/DoF = 2.9 × 10^4^) gave a K_d_ of 13.6 ± 1.5 nM for 0.94 ± 0.13 sites, ΔH = −7.2 ± 1.6 kcal/mol, ΔS = −1.7 cal/mol/deg. (B) In pull-down assays, GST-HNF1β bound only the E mutant of ΔIBB *Xenopus* Importin-α1 (that impairs NLS binding at the minor site) M_r_ 50 kD, but not the D mutant (that impairs binding at the major site) or ED double mutant (see Giesecke and Stewart, 2010).

Because the sequence of the HNF1β NLS is similar to that of classical monopartite NLSs (Lange et al., 2007, Marfori et al., 2011, Marfori et al., 2012), HNF1β is likely to be imported by Importin-α and to interact with its major NLS-binding site rather than being imported by direct binding to Karyopherin-β (Fontes et al., 2000). To test whether HNF1β and mImportin-α formed a complex, recombinantly expressed and purified HNF1β^DBD^ and mImportin-α1 in which the IBB domain had been deleted (ΔIBB-mImportin-α1) were mixed together and analysed by gel filtration chromatography (Fig. 3A), in which ΔIBB-mImportin-α1 and HNF1β^DBD^ co-eluted as a stoichiometric complex as observed by Coomassie blue stained SDS-PAGE analysis. To establish whether HNF1β interacts with other Importin-α isoforms, pull-down experiments using different ΔIBB-mImportin-α1 isoforms were performed (Fig. 3B). Crude extracts of GST-tagged HNF1β and ΔIBB-mImportin-α1 isoforms were mixed, then bound to glutathione resin, washed, and eluted by cleaving between GST and HNF1β^DBD^ using TEV protease. Both HNF1β^DBD^ and ΔIBB-mImportin-α1 were released, confirming that they were interacting (Fig. 3B). HNF1β interacted more strongly with ΔIBB-mImportin-α5 and less strongly with ΔIBB-mImportin-α3, -α4 and -α7 isoforms. The K_d_ for the interaction of ΔIBB-mImportin-α1 with the HNF1β NLS peptide was determined by isothermal calorimetry (ITC) (Fig. 4A) to be 13.6 ± 1.5 nM which was in the range commonly observed (Hodel et al., 2006). In summary, HNF1β binds at least two ΔIBB-mImportin-α isoforms strongly and some other isoforms more weakly.

Three different *Xenopus* Importin-α1 (*x*Importin-α1) mutants (Giesecke and Stewart, 2010) were used to define where the HNF1β NLS bound on Importin-α (Fig. 4B). These mutants contained point mutations that prevent binding via the major (D), minor (E), or both (ED) sites and also have the IBB domain removed to prevent auto-inhibition (Kobe, 1999). HNF1β^DBD^ bound to the E mutant but not the D or ED mutant of ΔIBB-*x*Importin-α1 (Fig. 4B), indicating that HNF1β binds primarily via the major site, consistent with its similarity to the SV40 NLS (Lange et al., 2007, Marfori et al., 2011, Marfori et al., 2012).

### 3.4 Structural characterisation of the interaction of mImportin-α1 with the HNF1β NLS

**Fig. 5:**
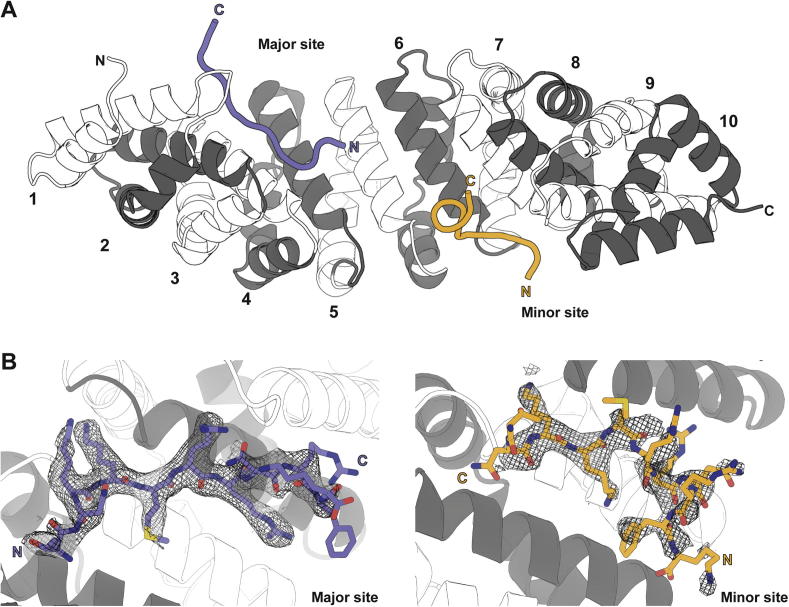
(A) Overview of the 2.4 Å crystal structure of the ΔIBB mouse Importin-α1:HNF1β^NLS^ complex. Two copies of the NLS peptide were observed where one was bound in the major site (blue) and another in the minor site (yellow). Each ARM repeat of mouse Importin-α1 has been labelled with alternating colours. (B) Final *2Fo-Fc* electron density map around NLS peptide in major and minor binding sites on mouse Importin-α1 contoured at 1σ.

To complement the functional and biochemical data, we also determined the 2.4 Å resolution crystal structure of the HNF1β NLS peptide bound to ΔIBB-mImportin-α1 (Fig. 5A). After initial rounds of refinement, strong difference density, corresponding to the peptide was found in both the major NLS-binding site on Importin-α1 together with weaker density at the minor site (Fig. 5B and C, respectively).

**Fig. 6:**
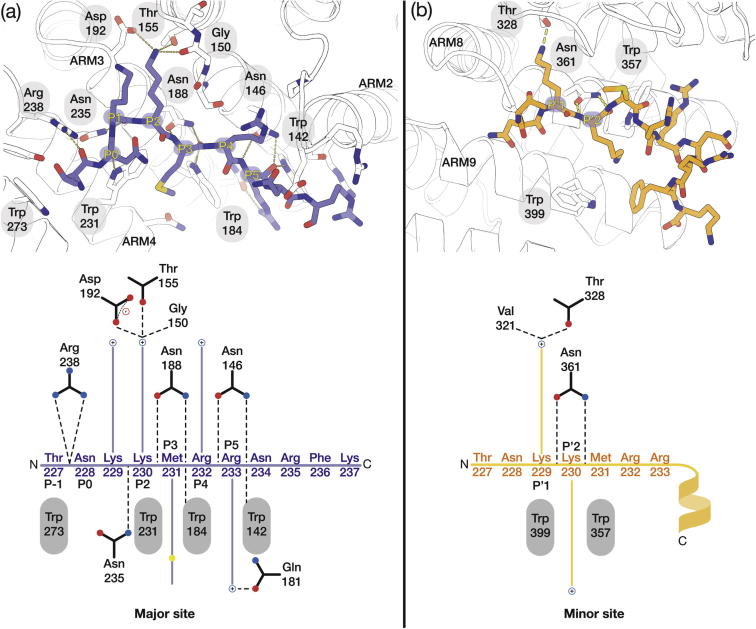
Schematic illustration of the interactions of the HNF1β^NLS^ peptide in the major (A) and minor (B) sites on ΔIBB mouse Importin-α1.

At the major site, the HNF1β NLS peptide backbone interacted with Importin-α1 via a set of conserved Asn residues on the import protein by H-bonding (Fig. 6A). The NLS residues are conventionally assigned to positions P1-P5 (Fig. 6). Key residues on Importin-α involved for the interaction with the NLS were Asn-235, 188 and 146, the latter two of which are involved in bidentate H-bonding with the NLS peptide backbone at P3 and P5. The aliphatic portions of the NLS peptide side chains were located in shallow pockets along the surface of Importin-α, but, as evident from Fig. 6A, the P2 residue, Lys230, forms many critical interactions, mostly by forming salt bridges with negatively charged residues on Importin-α. There were three interactions of the protonated nitrogen on the P2 lysine side chain with Asp192, Gly150 and Thr155. Residue P5 interacted with the side-chain of Gln181. Furthermore, NLS residues P3 and P5 were stacked against the aromatic indole rings of Trp231, Trp184 and Trp142 on Importin-α, with the latter two being aligned in parallel. Interestingly, residues ^231^MRRNR^235^ also form part of the HNF1β DNA binding site identified by crystallography (Lu et al., 2007; PDB accession code 2H8R), which is analogous to the overlap between NLS and DNA binding seen in the androgen receptor (Cutress et al., 2008; PDB accession code 3BTR).

Weaker difference density was also observed in the minor NLS-binding site on Importin-α1 and displayed a partly α-helical conformation (Fig. 6B). Several atypical, minor site selective NLSs have previously been characterised structurally to adopt a similar topology (Chang et al., 2013, Nakada et al., 2015). Our pull-down experiments with xImportin-α protein mutants have demonstrated that the interaction was primarily via the major site on Importin-α and so the presence of the peptide density in the minor site was probably due to the high protein concentration used to produce the crystals together with the peptide having additional degrees of freedom that may not present in the context of the intact HNF1β protein. NLS residues Lys229 (P1), Met231 (P3) and Arg233 (P5) bound into apolar and shallow pockets consisting of aromatic tryptophan residues (Trp231, Trp184 and Trp142) stacked in parallel.

Identifying the HNF1β NLS and its mode of interaction with Importin-α provides a basis of the design of therapeutic agents along the lines being investigated for other nuclear factors that aim to impair nuclear import to which cancer cells are often more sensitive (reviewed by Stelma et al., 2016). For example, Lin et al. (1995) developed a 41-residue synthetic peptide (that contained the NLS of transcription factor NF-κB together with a cSN50 cell membrane permeable motif) that inhibited the nuclear translocation of NF-κB and attenuated gene transcription in intact cells, but was not cytotoxic within the concentration range of the experiments (Torgerson et al. 1998).

In summary, we have confirmed, using eGFP-tagged deletion constructs, that in the HNF1β transcription factor that is overexpressed in ovarian clear cell carcinoma, the sequence ^229^KKMRRNR^235^ is essential for its nuclear localisation in transduced cell lines. The HNF1β^DBD^ was identified to interact with a spectrum of Importin-α isoforms by pull-down assays and we have further characterised the interaction between the putative NLS peptide and Importin-α using complementary biophysical techniques. Furthermore, we have determined the crystal structure of Importin-α in complex with the HNF1β NLS peptide to 2.4 Å resolution. This information should facilitate the development of compounds that target the nuclear import of the transcription factor. We have shown that HNF1β interacts strongly with Importin-α5 and selectively targeting this Importin-HNF1β interaction may open new avenues for the development of targeted therapeutics for ovarian clear cell carcinoma along the lines discussed by Stelma et al. (2016).

### 3.5 Data deposition

Co-ordinates and structure factors for the structure of the HNF1β peptides bound to Importin-α have been deposited at the Protein Data Bank (PDB) with accession code 5K9S.

## Figures and Tables

**Table 1:** Protein crystallography parameters for ΔIBB-mImportin-α1 complexed with the HNF1β NLS peptide.

*Data collection statistics*	
Wavelength (Å)	0.97624
Space group	*C222_1_*
Unit cell parameters: a, b, c (Å); α, β, γ (°)	81.4, 111.4, 129.4; 90.0, 90.0, 90.0
Resolution range (outer shell in brackets; Å)	129.4–2.40 (2.49–2.40)
Unique reflections	23,388 (2414)
Total observations	173,271 (18,283)
<I/σ(I)>: all (outer shell)	10.2 (1.8)
Rp.i.m.: all (outer shell)	0.041 (0.60)
Completeness: all (outer shell) (%)	100 (100)
Multiplicity	7.4
Wilson B-factor	53.7

*Refinement statistics*	
Non-hydrogen atoms	3500
Number of water molecules	53
Bond length deviation from ideal values (Å)	0.008
Bond angle deviation from ideal values (°)	0.78
Ramachandran favoured/outliers (%)	98.9/0
All-atom clashscore	1.86
Average protein B factor	67.2
Average water B factor	61.4
Rwork/Rfree (%)	18.4/22.0
MolProbity score (percentile)	0.95 (100)
