# Supplementary material for: Structural and calorimetric studies demonstrate that the hepatocyte nuclear factor 1β (HNF1β) transcription factor is imported into the nucleus via a monopartite NLS sequence
Source: J Struct Biol. 2016 Sep;195(3):273–81. doi: 10.1016/j.jsb.2016.06.018 (PMC4991853; doi:10.1016/j.jsb.2016.06.018)
Supplement: Supplementary data [file mmc1.docx]

**Supplementary Information**

***HNF1β localisation studies in IOSE4 and JHOC9 cell lines***

The localisation pattern of HNF1β was studied using eGFP fusions of HNF1β, HNF1α and isolated eGFP in previously constructed transduced normal ovarian surface epithelial 4 (IOSE4) cell line [52]. Untransduced IOSE4 was used as the negative control. HNF1α and HNF1β eGFP fusions localised in the nucleus (Figure S1A and B). Nuclear and cytoplasmic localisation was observed for control cells expressing eGFP alone (Figure S1C). Untransduced control line IOSE4 did not display any noticeable levels of autofluorescence (Figure S1D).

Localisation of endogenous HNF1β was also studied in CCC line JHOC9 as a more disease-specific model using IF using anti-HNF1β SAB1406512 antibody (Figure S2A). Non-specific binding of the secondary antibody and autofluorescence were not observed (Figure S2B and C). Non-specific binding of primary antibody was also not observed for SAB1406512 (Figure S2D).


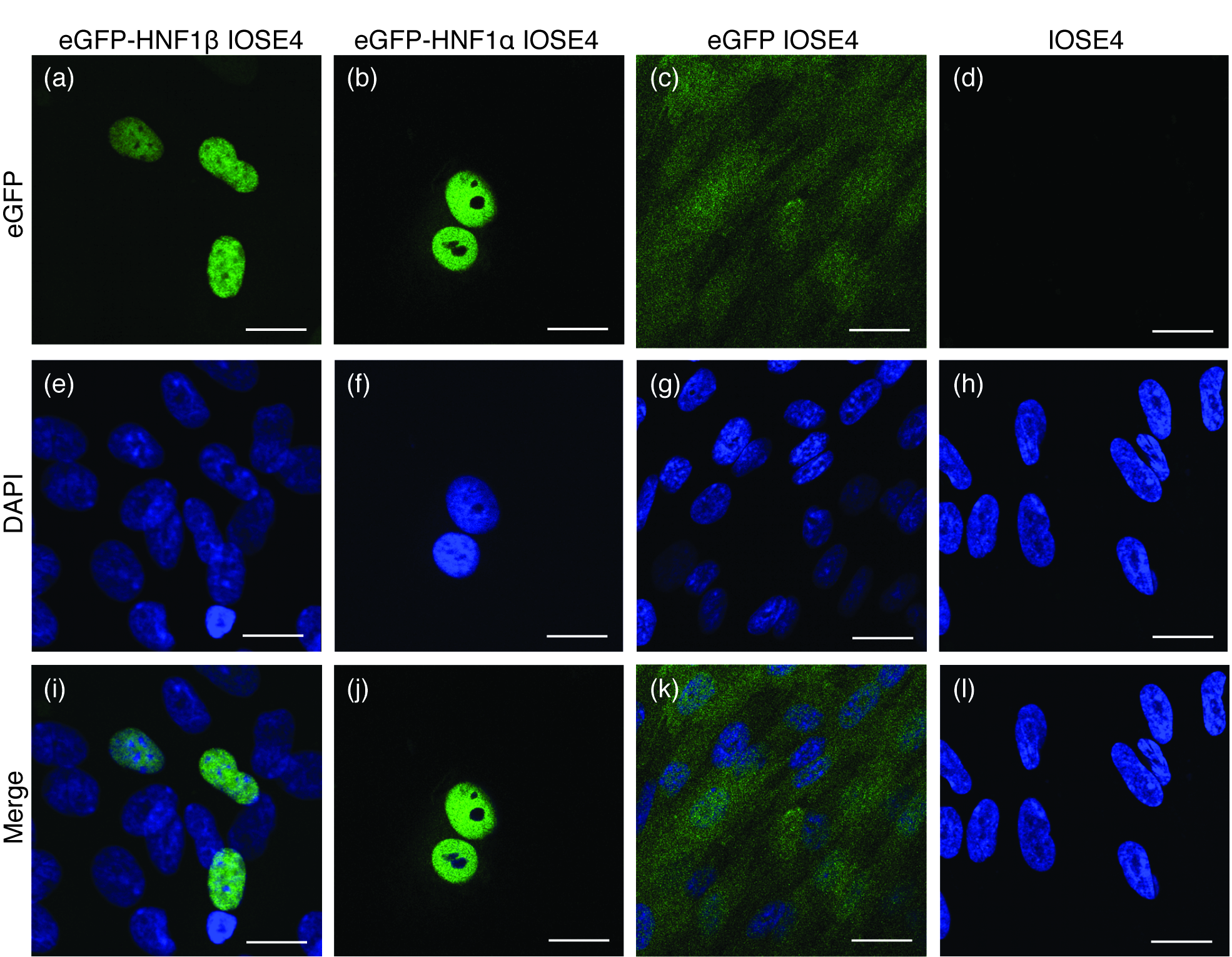


**Supplementary Figure S1** – Subcellular localisation of eGFP-tagged wild-type HNF1α and HNF1β proteins in transduced IOSE4 cell lines. A, E, I: eGFP-HNF1β IOSE4; B, F, J: eGFP-HNF1α IOSE4; C, G, K: eGFP IOSE4; D, H, L: IOSE4. Cells were fixed and stained with DAPI. Images were taken on a Leica tandem confocal microscope using a 60 x objective. The scale bar in each image represents 40 μm.


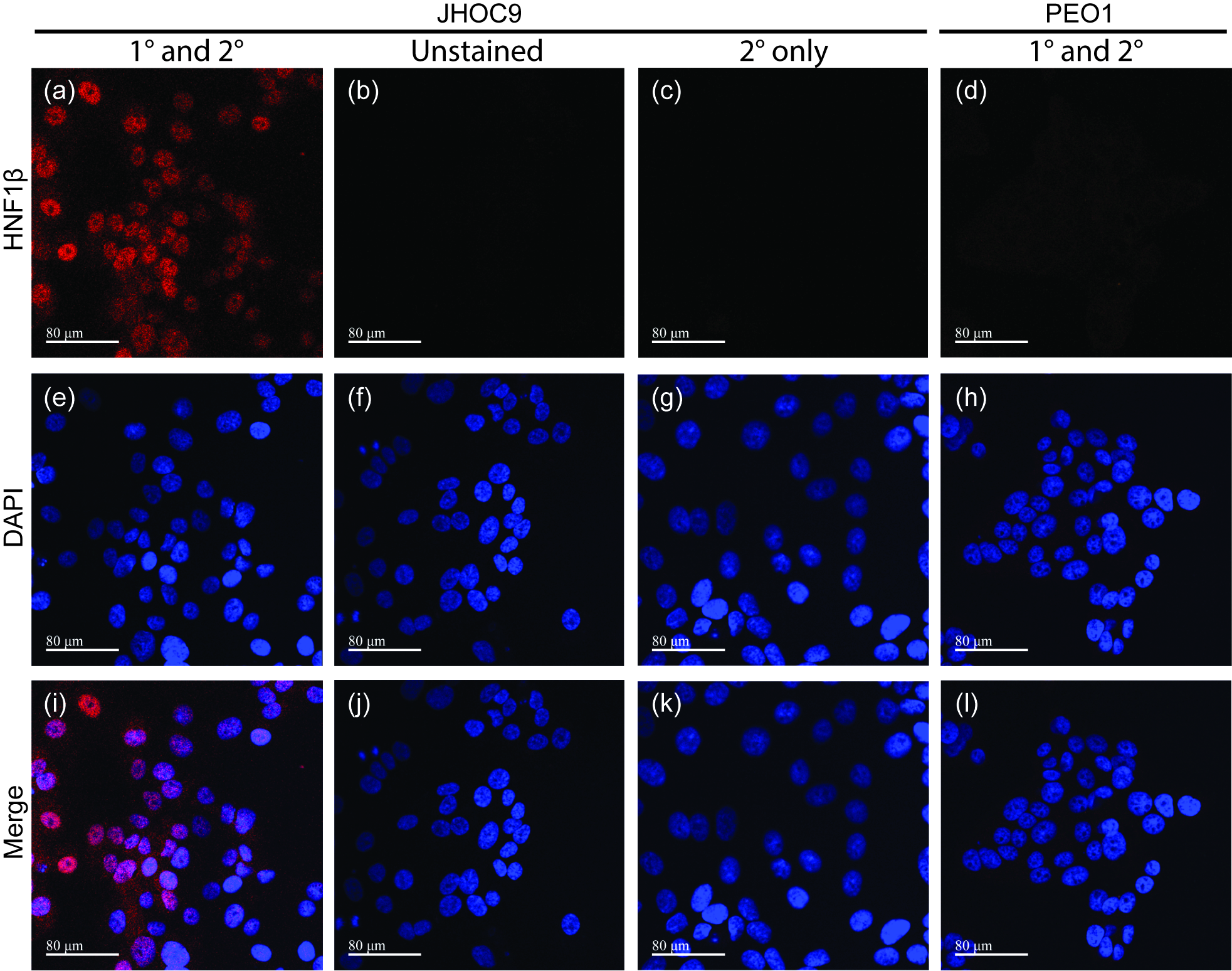


**Supplementary Figure S2** - Optimised Immunofluorescence staining of JHOC9 line with anti-HNF1β SAB1406512 antibody (1:300 dilution). PEO1 line was used as a negative control (SAB1406512, 1:300 dilution). The scale bar represents 80 μm. Images were taken on a Leica tandem confocal microscope using a 40 x objective.
